# Editorial: Physiological performance of aquatic animals under farming-induced stress conditions

**DOI:** 10.3389/fphys.2023.1136611

**Published:** 2023-01-16

**Authors:** Abdallah Tageldein Mansou, Mohamed Ashour, Cristóbal Espinosa Ruiz, Neeraj Kumar, Maria Angeles Esteban

**Affiliations:** ^1^ Animal and Fish Production Department, College of Agricultural and Food Sciences, King Faisal University, Al-Ahsa, Saudi Arabia; ^2^ Fish and Animal Production Department, Faculty of Agriculture (Saba Basha), Alexandria University, Alexandria, Egypt; ^3^ National Institute of Oceanography and Fisheries, Cairo, Egypt; ^4^ ICAR-National Institute of Abiotic Stress Management, Ministry of Agriculture, Govt of India, Baramatio, India; ^5^ Cell biology and histology, Faculty of biology, Murcia University, Murcia, Spain

**Keywords:** physiological response, stress, aquaculture, environment, pollution

One of the newest industries, aquaculture is expanding to offset the sharp rise in demand for aquatic products (FAO, 2020; Mansour et al., 2022). As a result, new methods have been devised to boost sustainable aquaculture production in opposition to climatic changes and pollution (Ahmed et al., 2020). Examples of these include recirculatory aquaculture systems, aquaponics, integrated farming, nano-feed technology, nutrigenomics application, compensatory growth technology, smart and precisive aquaculture, and biofloc technologies, etc (O'Donncha and Grant, 2019; Costa-Pierce, 2021).

However, most aquaculture systems are used at high or extremely high stocking densities, which eventually leads to a rise in the incidence of biotic and abiotic stresses on cultured aquatic animals (Abisha et al., 2022). High stocking density, ammonia levels, and microbial load, low dissolved oxygen, and species-specific interactions are a few examples of these stressors. Due to these rearing conditions, aquatic animals run the risk of experiencing a number of physiological reactions, including dysregulated hemostasis, oxidative stress, low growth, poor feed conversion ratio, immune suppression, disease susceptibility, and finally, a high mortality rate (Balasch and Tort, 2019; Petitjean et al., 2019). Unfortunately, little is known about the physiological reactions of aquacultured animals to the use of various rearing techniques and stressors at this time.

Researchers have contributed to this Research Topic to help us better grasp how modern farming methods affect aquatic organisms’ physiological function. This evaluation is crucial to ensure both the viability of these contemporary systems and the provision of sustainable environmental conditions that will ultimately improve fish health and production.

The study of Liu et al. showed that increasing stocking density of Greater Amberjack (*Seriola dumerili*) during assimilated transportation for 8 h induced an increasing tendency of cortisol and significantly increased catalase and glutathione peroxidase levels. In addition, the expression levels of immune-related factors were significantly decreased with increasing stocking densities. These physiological changes were associated with water quality deterioration, including decreased pH and ammonia nitrogen, and nitrite increase.

For more extend of water quality effects on physiological pe rformance Huang et al. evaluated the response of large-scale loach (*Paramisgurnus dabryanus*) to different alkalinity levels and durations on the expression of some osmoregulatory genes. When large-scale loach exposed to high alkalinity, the fish upregulates the Rhesus glycoproteins (Rhag and Rhcg) to help NH_3_ efflux from the gills. Aquaporins-1 transcription in the gills in order to excrete excess internal water, and downregulation of aquaporins-3 in order to block urea elimination together maintain appropriate osmolality as an adaptation to alkaline environments.

Hypoxia is considered one of the most aquaculture common threats. The physiological response of rainbow trout to acute hypoxia exposure was studied in different tissues including the brain, skin, and head kidney García-Meilán et al. The findings demonstrated that all biological systems, including neuroendocrine, metabolism, and immune, contribute in the regulation of the response and the recovery process, regardless of the kind of stressor.

A more invasive approach was applied by Sayed et al. for determining the physiological response of *Clarias gariepinus* to water pollution with dexamethasone. The exposure to dexamethasone significantly reduced hematological performance, disrupt antioxidant status, decreased acetylcholinesterase activity, and increased cortisol levels. Vital organs function were significantly deteriorated with dexamethasone exposure and inflammatory cytokine (IL-1β and IL-6) expression was upregulated, which reflects the deleterious physiological effects of dexamethasone.

Finally, Ghori et al. presented the role of dietary supplementation of probiotics in individuals or a combination with *Bacillus cereus* and *Geotrichum candidum* on growth, physiological status, and challenge by *Staphylococcus aureus*. The probiotic supplementation improved growth, feed utilization, hematological profile, and digestive enzyme activities as compared to the control. The survival rate was higher in groups fed combined probiotics and challenged with *S. aureus*. Fish gut microbial composition was driven by dietary probiotics whereas opportunistic pathogens were eliminated such as *Staphylococcus saprophyticus* and *Sporobolomyces lactosus*, and low levels of *Trichosporon* and *Cryptococcus* were detected.
